# Selenoprotein P concentrations and risk of progression from mild cognitive impairment to dementia

**DOI:** 10.1038/s41598-023-36084-6

**Published:** 2023-05-31

**Authors:** Marco Vinceti, Teresa Urbano, Annalisa Chiari, Tommaso Filippini, Lauren A. Wise, Manuela Tondelli, Bernhard Michalke, Misaki Shimizu, Yoshiro Saito

**Affiliations:** 1 Department of Biomedical, Metabolic, and Neural Sciences, CREAGEN - Environmental, Genetic, and Nutritional Epidemiology Research Center, University of Modena and Reggio Emilia, Modena, Italy; 2 Department of Biomedical, Metabolic, and Neural Sciences, Center for Neurosciences and Neurotechnology, University of Modena and Reggio Emilia, Modena, Italy; 3 Department of Epidemiology, Boston University School of Public Health, Boston, MA USA; 4 Neurology Unit, University Hospital, Modena, Italy; 5 School of Public Health, University of California Berkeley, Berkeley, CA USA; 6 Primary Care Department, Local Health Unit of Modena, Modena, Italy; 7 Research Unit Analytical BioGeoChemistry, Helmholtz Center Munich German Research Center for Environmental Health GmbH, Neuherberg, Germany; 8 Laboratory of Molecular Biology and Metabolism, Graduate School of Pharmaceutical Sciences, Tohoku University, Sendai, Japan

**Keywords:** Cognitive neuroscience, Diseases of the nervous system, Learning and memory, Molecular neuroscience, Dementia, Neurodegeneration, Risk factors

## Abstract

There is a growing literature investigating the effects of selenium on the central nervous system and cognitive function. However, little is known about the role of selenoprotein P, the main selenium transporter, which can also have adverse biological effects. We conducted a prospective cohort study of individuals aged 42–81 years who received a clinical diagnosis of mild cognitive impairment. Using sandwich ELISA methods, we measured full-length selenoprotein P concentrations in serum and cerebrospinal fluid to assess the relation with dementia incidence during a median follow-up of 47.3 months. We used Cox proportional hazards regression and restricted cubic splines to model such relation. Of the 54 participants, 35 developed dementia during follow-up (including 26 cases of Alzheimer’s dementia). Selenoprotein P concentrations in serum and cerebrospinal fluid were highly correlated, and in spline regression analyses they each showed a positive non-linear association with dementia risk, particularly after excluding dementia cases diagnosed within 24 months of follow-up. We also observed differences in association according to the dementia subtypes considered. Risk ratios of dementia peaked at 2–6 at the highest levels of selenoprotein P, when compared to its median level, also depending on matrix, analytical methodology and dementia subtype. Findings of this study, the first to assess selenoprotein P levels in the central nervous system in vivo and the first to use a prospective study design to evaluate associations with dementia, suggest that higher circulating concentrations of selenoprotein P, both in serum and cerebrospinal fluid, predict progression of MCI to dementia. However, further confirmation of these findings is required, given the limited statistical precision of the associations and the potential for residual confounding.

## Introduction

The involvement of selenium and particularly selenoprotein P, one of the selenium-containing proteins, in the etiology of human disease is unclear and controversial^1–4^. Selenoprotein P is a major plasma selenoprotein containing selenocysteine residues and encoded by the SELENOP gene using a UGA stop codon^5,6^. This selenoprotein shares with its cofactor, the metalloid selenium, the fate of being advocated as both beneficial and detrimental, with a still not well defined but certainly narrow safe range of biological activity^7–10^. Selenoprotein P has a physiological and nutritional role as a selenium transporter and antioxidant due to its capacity to reduce phospholipid hydroperoxide^11–13^, but it may have adverse metabolic effects in humans^10^. Excess selenoprotein P concentrations have been implicated in the etiology of type 2 diabetes^14–16^, pulmonary hypertension^17–19^, non-alcoholic fatty liver disease^20–22^, and other conditions^16,23–25^. However, while knowledge of its metabolic properties has progressed, little is known about its involvement in the etiology of neurodegenerative disease in humans, despite a growing number of animal and laboratory studies on this topic^4,26^. In a recent cross-sectional study, the highest concentrations of selenoprotein P in serum were associated with a trend towards decreased serum β‑amyloid content, a biomarker suggesting a higher risk of neurodegeneration^27^. However, few human studies were conducted in vivo focusing on selenoprotein P the in the central nervous system, apart from a recent one assessing the relation of selenoprotein P in cerebrospinal fluid and blood with biomarkers of neurodegenation in patients with Alzheimer’s dementia^28^, and from a few studies carried out on autoptic samples^29–31^. This is particularly relevant since circulating levels of selenium and selenoprotein P may not reflect its central nervous system content, as shown by a speciation study in paired samples of cerebrospinal fluid and serum from the same patients^32^. In addition, few studies to date have used a cross-sectional design, precluding assessment of the temporality of the associations and hampering a causal interpretation of the results, particularly when samples from affected individuals were used. In fact, the behavioral changes in cases (including the altered nutritional status) and the metabolic effects of the neurodegenerative process particularly in the central nervous system, could affect selenium status and therefore induce a reverse causation bias.

In this cohort study, we ascertained baseline biomarkers of selenoprotein P in cerebrospinal fluid and blood of subjects with mild cognitive impairment (MCI) and assessed the extent to which these biomarkers were related to subsequent progression to dementia.

## Results

In Table 1, we describe baseline demographic characteristics of the study population and related selenoprotein P concentrations. Such concentrations were higher when using the BD1 assay as compared with AA3, indicating the presence of N-terminal fragment of selenoprotein P. Concentrations were higher among males and older individuals, while no association emerged with education.

**Table 1:** Baseline characteristics along with median (50th) and interquartile range (IQR) concentrations of selenoprotein P (AA3 and BD1) as ng/mL in cerebrospinal fluid (CSF) and CSF selenoprotein P-bound selenium levels from a previous study^43^ as µg/L for 54 individuals affected by mild cognitive impairment.

Characteristics	N	%	CSF		Serum		Selenoprotein P-bound selenium in CSF
			AA3	BD1	AA3	BD1	
			50th (IQR)	50th (IQR)	50th (IQR)	50th (IQR)	50th (IQR)
All participants	54	100	28.7 (19.8–36.1)	76.0 (56.0–94.0)	6952.0 (6191.0–7686.0)	10,999.3 (9868.6–11,939.5)	1.58 (1.14–2.04)
Sex							
Men	28	51.9	30.6 (21.8–38.7)	81.5 (59.5–100.0)	6940.0 (6075.0–7841.0)	11,583.7 (9776.6–12,098.4)	1.68 (1.15–2.09)
Women	26	48.1	26.3 (16.9–32.5)	67.0 (36.0–86.0)	6952.0 (6198.0–7406.0)	10,886.8 (10,198.5–11,538.9)	1.33 (0.86–1.80)
Age (years)							
< 65	24	44.4	27.9 (18.2–36.4)	66.0 (32.5–96.0)	7062.0 (6198.0–7731.0)	10,944.0 (10,016.5–11,893.6)	1.63 (1.17–1.96)
≥ 65	30	55.6	29.4 (21.7–35.9)	80.0 (56.0–93.0)	6769.0 (6191.0–7406.0)	11,017.6 (9823.1–11,785.6)	1.52 (0.96–2.11)
Education (years)							
< 8	18	33.3	30.6 (22.4–33.1)	82.5 (56.0–101.0)	7231.0 (6187.0–8483.5)	11,008.4 (9822.6–11,992.9)	1.33 (0.96–1.97)
≥ 8 and ≤ 12	15	27.8	29.6 (13.4–39.2)	75.0 (9.0–93.0)	6628.0 (6191.0–7731.0)	11,354.5 (10,172.8–11,694.5)	1.87 (0.68–2.53)
≥ 13	21	38.9	27.2 (19.8–33.6)	75.0 (56.0–94.0)	6883.0 (6198.0–7114.0)	10,665.0 (9885.9–11,689.2)	1.63 (1.18–1.78)

**Figure 1:**
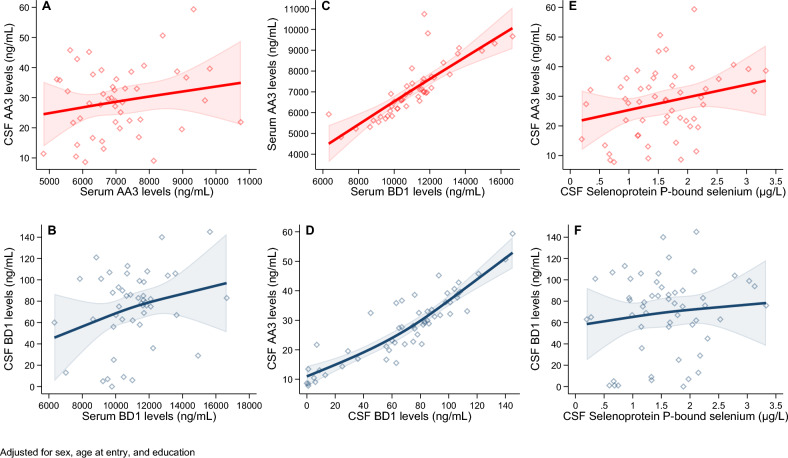
Spline regression analysis for the association adjusting for sex, age, and education in 54 individuals with mild cognitive impairment between concentrations in cerebrospinal fluid (CSF) and in serum of AA3 (**A**) and BD1 (**B**), between serum concentrations of AA3 and of BD1 (**C**) and CSF concentrations of AA3 and of BD1 (**D**), and between CSF selenoprotein P-bound selenium and CSF AA3 (**E**), and CSF selenoprotein P-bound selenium and CSF BD1 (**F**).

In linear and non-linear spline regression analyses (Figs. 1 and S1), the AA3 and BD1 assays were positively and almost linearly correlated within both matrices, with roughly comparable magnitude and precision of the estimates. With reference to the paired analysis of serum and CSF samples of the same biomarker, values yielded by the BD1 and AA3 assays were positively correlated despite limited precision of the association, the former biomarker showing a steeper curve of the relation. CSF concentrations of selenoprotein P-bound selenium were positively associated with CSF selenoprotein P concentrations independently from the assay used, though the association with full-length protein (AA3) was more precise.

Duration of follow-up was 51.1 months on average, with a median of 47 months (interquartile range-IQR 26–70). Of the 54 participants diagnosed with MCI at baseline, 19 did not convert at the follow-up visit, while 26 progressed to Alzheimer’s dementia, 5 to frontotemporal dementia, 2 to dementia with Lewy bodies, and 2 to vascular dementia. Table S1 shows median and IQR concentrations of CSF β-amyloid, total and phosphorylated Tau according to diagnosis at follow-up. MCI subjects converting to Alzheimer’s dementia exhibited lower levels of β-amyloid compared to MCI individuals converting to other dementia, which in turn had lower levels compared to MCI not converting. Opposite results were observed for phosphorylated and total Tau, with higher levels encountered in MCI participants converting to Alzheimer’s dementia. In Fig. S2 we report boxplots for BD1 and AA3 CSF (A) and serum (B) levels according to diagnosis at follow-up. We then analyzed the relation between AA3 and BD1 and amyloid beta 42/40, phospho-Tau181 and total Tau concentrations in linear and spline regression models (Figs. S3–S6).

**Figure 2:**
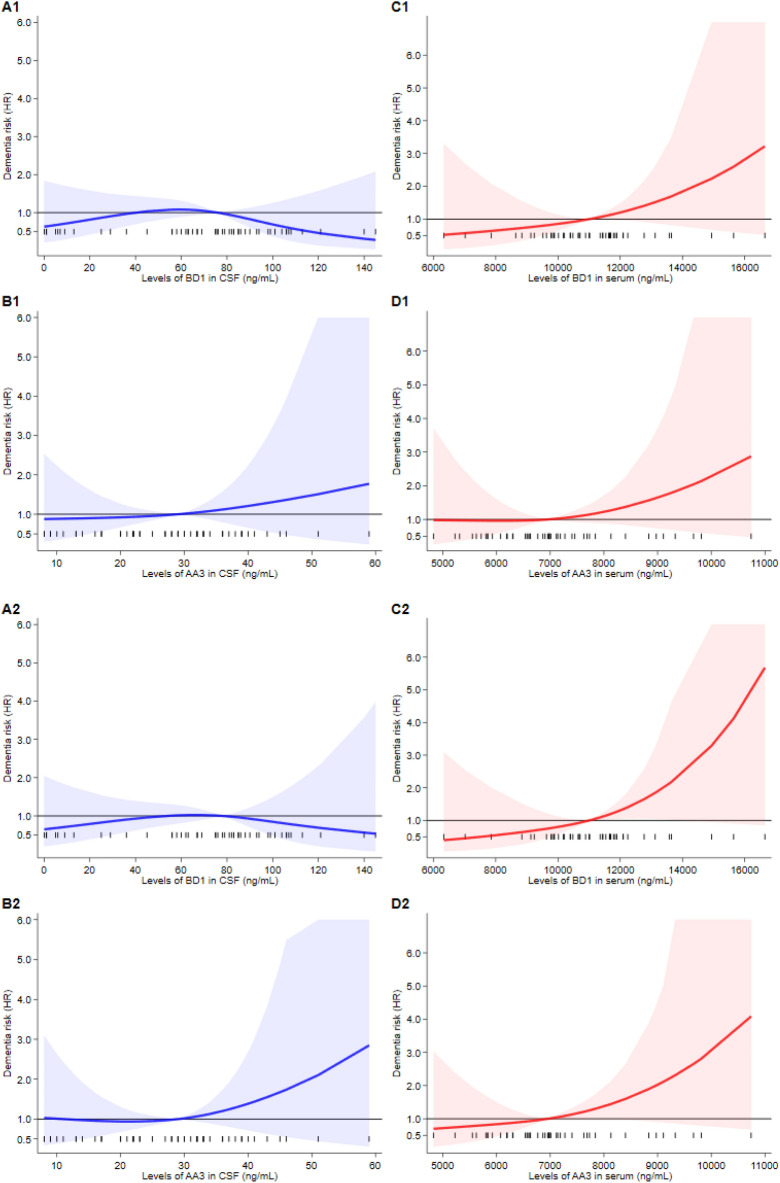
Restricted cubic spline analysis of Cox proportional hazards model for the association between baseline selenoprotein P concentrations and risk of developing dementia (all dementia combined). Overall analysis (**A1**–**D1**) and with the exclusion of participants who progressed to dementia within 24 months from the first visit (**A2**–**D2**). The solid line represents the multivariable hazard ratio (adjusted by sex, age, and education) with upper and lower confidence interval showed by shaded area.

**Figure 3:**
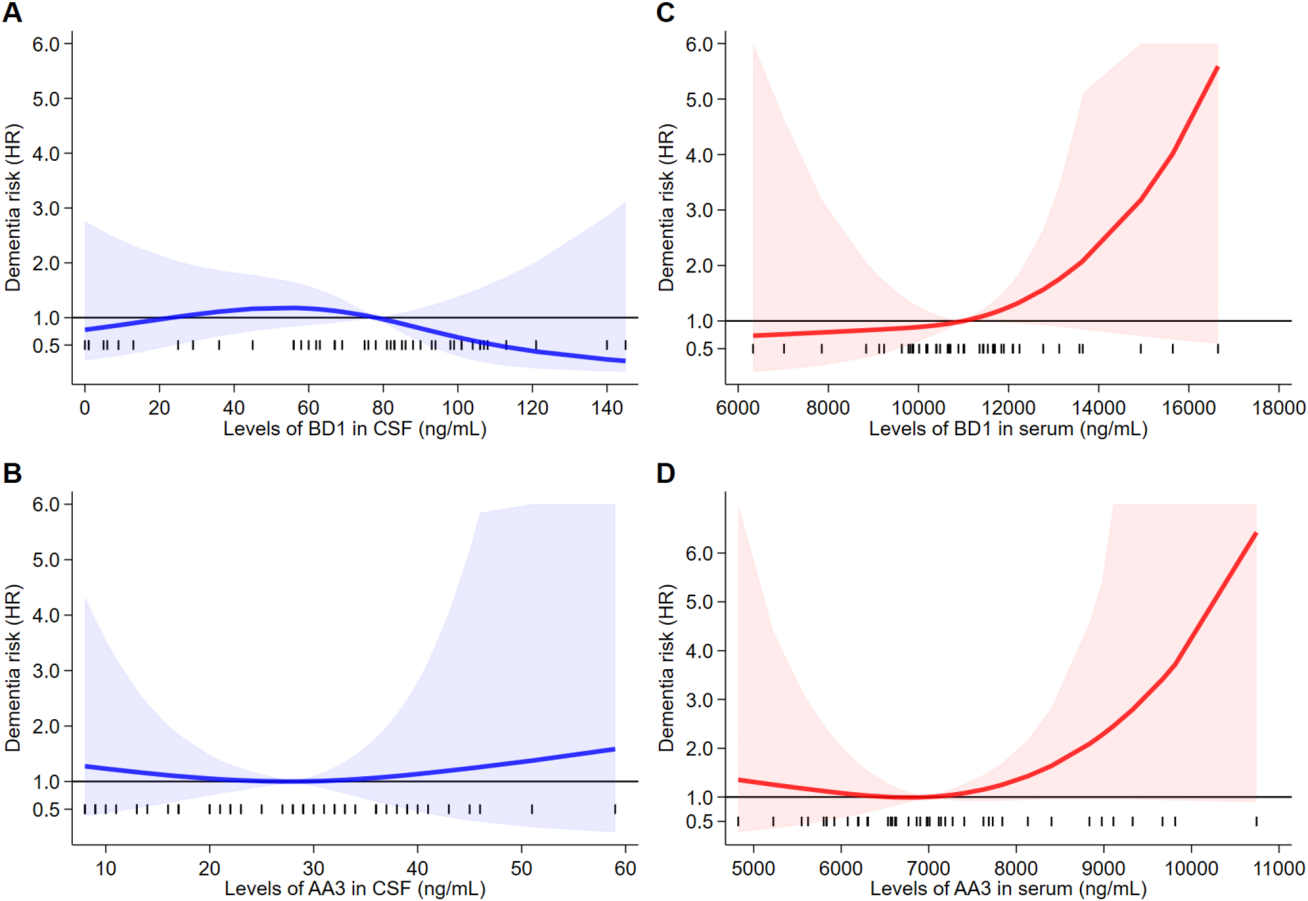
Restricted cubic spline analysis of Cox proportional hazards model for the association between baseline selenoprotein P concentrations and risk of developing Alzheimer’s dementia, based on the 26 participants who progressed to dementia after the first 24 months of follow-up (**A**–**D**). The solid line represents the multivariable hazard ratio (adjusted by sex, age, and education) with upper and lower confidence interval showed by shaded area.

**Figure 4:**
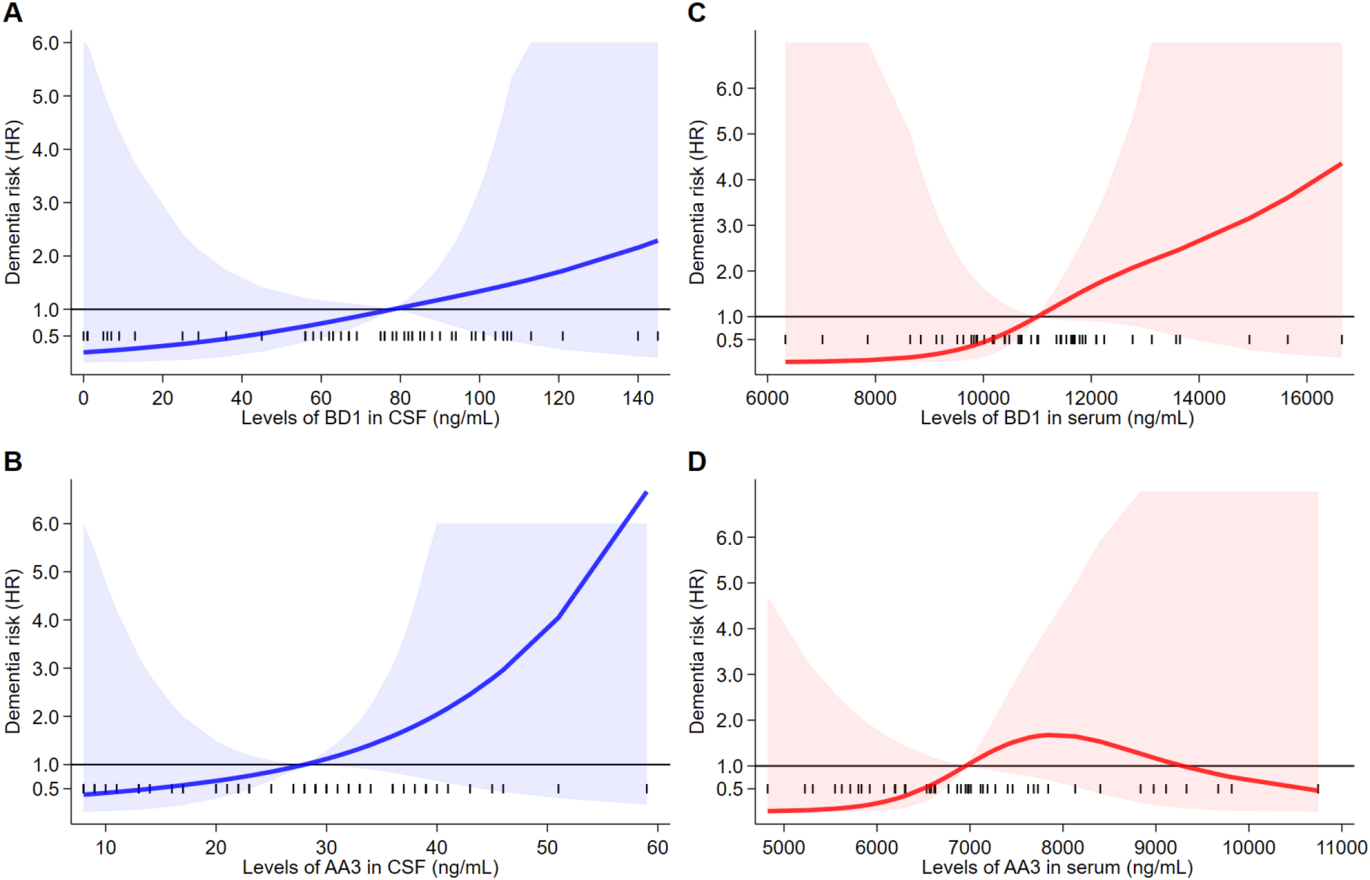
Restricted cubic spline analysis of Cox proportional hazards model for the association between baseline selenoprotein P concentrations and risk of developing dementia of non-Alzheimer’s type (**A**–**D**), n = 9. All cases occurred after 24 months of follow-up from baseline. The solid line represents the multivariable hazard ratio (adjusted by sex, age at entry, and education) with upper and lower confidence interval showed by shaded area.

Results of the Cox regression analyses for any dementia outcome adjusted for covariates are reported in Fig. 2. In CSF, BD1 concentrations were not associated with increased risk of dementia, while selenoprotein P concentrations based on the AA3 assay were moderately and positively associated with dementia risk above the reference point of 30 ng/mL, with an almost linear pattern above this cutpoint. When considering the serum concentrations of selenoprotein P, values yielded by both the AA3 and BD1 assays were associated with increased risk of progression to dementia above their reference point (10,999 and 6952 ng/mL, respectively), with a steeper increase at the highest concentrations of both BD1 and AA3 (Fig. 2A1–D1). When we did not consider the dementia cases that occurred during the first two years of follow-up, we found similar trends for the serum levels of both AA3 and BD1 in blood, even if with a much steeper non-linear increase for the latter, and a non-linear increase in dementia risk for CSF AA3 above the cutpoint (Fig. 2A2–D2). Results changed little when we restricted cases to Alzheimer’s dementia also excluding dementia cases diagnosed in the first two years of follow-up, except for a weaker positive association between the CSF AA3 concentrations above the threshold and disease risk and for a stronger association between serum AA3 concentration and risk (Fig. 3A–D). In addition, when the model was also adjusted for APOE status, an increased risk was observed for CSF AA3 concentrations compared to the overall Alzheimer’s dementia analysis (Fig. S7). When the case group was restricted to dementia not due to Alzheimer’s disease (Fig. 4), we observed a clear positive association with dementia risk above the reference point for both BD1 and AA3 in CSF and for BD1 in serum, but no association between serum AA3 and risk. In this subgroup, positive associations between BD1 and risk were roughly linear for both CSF and serum concentrations, though the positive associations with dementia above the threshold of the biomarker was steeper when serum concentrations were considered. Conversely, in CSF, the association between the AA3 selenoprotein form and dementia risk was flat below the median but steeply and non-linearly increased above that threshold.

When we excluded from all the aforementioned analyses the two cases of vascular dementia that occurred during follow-up (both diagnosed later than 24 months from the baseline), or the two cases of type 2 diabetes already existing at baseline (one of which progressed to Alzheimer’s dementia), there was little change in the pattern or magnitude of the risk estimates.

## Discussion

In this first prospective study of circulating (blood and CSF) concentrations of selenoprotein P and dementia risk, greater concentrations were generally predictive of higher risk, particularly when exposure assessment was based on the full-length protein assay AA3. In particular, positive associations emerged based on the measurement of blood concentrations, while a positive association based on CSF concentrations was observed only after removing dementia cases diagnosed early in follow-up. This indicated the possibility of reverse causation as an explanation of our findings when considering the overall study population, due to the effect of the pathological process underpinning the progression of dementia that in the proximity of the clinical onset of the disease may affect selenoprotein P levels. Such potential for bias in studies addressing selenium and selenoproteins in relation to dementia risk has been documented previously, particularly for studies based on prevalent cases or post-mortem tissues^29,31,33,34^. In our study, risk of an early effect of dementia progression on selenoprotein P concentrations appeared to be particularly high for CSF concentrations compared with blood concentrations, and therefore the former might yield valid values for disease prediction long before dementia onset, while becoming of limited predictive value when dementia diagnosis is approaching. Overall, our findings suggest that dementia should also be considered when dealing with the adverse effects of selenoprotein P, in addition to metabolic and vascular endpoints ascribed to selenoprotein P excess^9,10^. In particular, there is a need for prospective studies in humans to assess the role of selenoprotein P in dementia etiology, possibly assessing its concentrations in the central nervous system through its cerebrospinal fluid content and encompassing a satisfactorily longer period of follow-up.

We found a positive association between concentrations of selenoprotein P (independent of the assay used) in blood and in CSF, which represents a novel finding. The consistency of results was unexpected, given that some selenium species (including selenoprotein P-bound selenium) have shown weak correlations in paired analyses between these two compartments^32^. We also found a strong correlation between the results yielded by the two assays AA3 and BD1 in serum and CSF, that nevertheless could not prevent one sets of results (BD1) from predicting conversion to dementia differently than the other. This further supports the use of the assay for the full-length protein in determining selenoprotein P, consistent with most epidemiologic studies of this selenoprotein^14^.

The predictive effects of high circulating concentrations of selenoprotein P in blood and to a lower extent in CSF may indicate either a predictive role of this selenium-containing protein for conversion to dementia or a direct causal role in the progression to the disease from MCI. In the first instance, the positive association would only reflect unmeasured confounding by risk factors causally related to dementia etiology and onset. Under this scenario, selenoprotein P concentrations could covariate with the causal factors for still unknown metabolic and pathological reasons, or could reflect a compensatory response of this selenoprotein to oxidative stress, due to the need to increase selenium transport and availability or to a direct antioxidant role of the protein^10,11,26,35,36^. Such a compensatory effect of oxidative stress and Alzheimer’s disease has been suggested following analyses of post-mortem brain samples^29,31^, in which high concentrations of selenoprotein P lesions were interpreted as a defense response to the local oxidative stress characterizing the pathologic process^29^. However, such a hypothesis is not supported by our findings, since the positive relation between selenoprotein P concentrations and dementia risk was stronger when we excluded subjects diagnosed in the first period of follow-up, despite such patients were those for which a stronger effect of oxidative stress in raising selenoprotein P levels would have expected. In addition, and alternatively to the aforementioned hypothesis, increased expression of selenoprotein P with proteolysis could possibly induce organic selenium deposits and potentiate Alzheimer’s pathology and heighten risk of dementia^29^. The possibility of an etiologic role of selenoprotein P in dementia etiology would be consistent with previous evidence from the few human studies available, such as the positive association observed between high brain selenium content and features of Alzheimer’s disease in autoptic studies^33^, and the inverse association between the highest levels of selenoprotein P and lower levels of serum β-amyloid, a marker of increased Alzheimer’s disease risk^27^.

If not due to unmeasured confounding, our results indicate a possible adverse effect of high concentrations of selenoprotein P in dementia etiology, in line with deleterious effects recently ascribed to selenoprotein P with reference to metabolic disease risk^8–10,18,37^. As largely expected for a nutrient such as selenium and its selenoproteins, such deleterious effects favoring conversion of MCI to dementia would occur only at high concentrations of this selenoprotein, mirroring the more general U-shaped effect of this essential element on human health^7,10^. In addition, the growing evidence suggesting an etiologic role of insulin resistance and glucose metabolism abnormalities in the etiology of dementia and specifically of Alzheimer’s disease^38–41^ provides biological plausibility for involvement of selenoprotein P in dementia etiology, given the adverse effects on insulin sensitivity and more generally glucose metabolism induced by this selenoprotein^13,15,37,42^.

In a previous study in this cohort, we did not find an association between baseline CSF levels of selenoprotein P-bound selenium and dementia risk^43^, which was instead positively associated with inorganic hexavalent selenium (selenate). Such differences could be due to the longer period of follow-up in the present study with consequent reduction of reverse causation bias, or to the differential use of selenoprotein P-bound selenium in the previous study^43^ and the direct selenoprotein assay in the present one, given that each molecule of this protein may contain a variable number of selenium atoms (as selenocysteine residues)^44–49^, as also suggested by the limited positive relation between concentrations of selenoprotein P and of selenoprotein P-bound in CSF. The assessment of the correlation between selenoprotein-P concentrations and selenoprotein P-bound selenium in this study in CSF has not been previously performed, and the scattered distribution of the observations within our population is therefore a novel finding that warrants confirmation in future studies.

Our subgroup analyses according to dementia types indicate that a role of selenoprotein P in predicting dementia conversion in individuals with MCI could be more pronounced for the forms not induced by Alzheimer’s dementia, such as frontotemporal dementia and Lewy bodies dementia. Those clinical forms are ascribed to different pathologic processes probably induced by different risk factors, and it is conceivable that the involvement of a detrimental effect of too high levels of selenoprotein P could be more relevant in triggering these mechanisms compared with the β-amyloid plaques and neurofibrillary tangles deposition, the pathologic hallmarks of Alzheimer’s disease^50,51^. In addition, our findings suggest that when assessing the relation between selenoprotein P concentrations and incidence of non-Alzheimer’s dementia, it is necessary to rely on CSF concentrations of the biomarker rather than its blood levels, differently from what appears to be true for Alzheimer’s dementia.

Study limitations include its limited sample size, which reduced the precision of the risk estimates and the certainty of our results as shown by the wide confidence intervals, especially in subgroup analysis. An additional limitation that is related to outcome assessment is the lack of neuropathological examinations *post-mortem*, due to the fact that few cohort members died during follow-up and that such autoptic assessment is very unlikely to occur. However, we based our diagnosis of dementia on both clinical and biochemical evidence, the latter being CSF biomarkers of neurodegeneration and amyloidosis, which have high sensitivity and specificity for detecting dementia due to Alzheimer’s disease relative to neuropathological *post-mortem* assessment^52^. Finally, we must acknowledge the potential for residual confounding inherent in the observational study design.

Despite these limitations, we found comparable risk estimates when we excluded two cases of vascular dementia, likely having different etiology compared with the other neurodegenerative forms of the disease. However, a larger study population would be needed to get more precise risk estimates and to detect small changes in the relative risks of the single dementia subtypes. Exclusion of diabetic cases at baseline also did not affect the estimates, a finding increasing the validity of our findings given the relation between high selenoprotein P levels and altered glucose metabolism^8,13^.

In conclusion, results from this first in vivo prospective cohort study, suggest that concentrations of two biomarkers of full-length selenoprotein P levels in serum and CSF predict conversion towards dementia among individuals with MCI. Thus, high levels of selenoprotein P, a protein that appears to exert both beneficial and toxic effects also depending on its dose, might have deleterious effects on cognitive function, though caution should be used in drawing such a conclusion given the aforementioned study limitations. Given the relevance of selenium metabolism in the brain^4,53^, the limited evidence available on the involvement of selenoprotein P in the etiology of neurological diseases in humans, and the inability of peripheral indicators (such as serum) of selenoprotein P status in relation to its central nervous system concentrations, further in vivo human studies assessing the relation between brain selenoprotein P and dementia risk are clearly warranted.

## Methods

### Study population

During 2008–2014, we recruited 56 individuals who received a clinical diagnosis of MCI at the Neurology Memory Clinic of Policlinico University Hospital in Modena, Northern Italy^43^. All participants underwent lumbar puncture and venepuncture for diagnostic purposes. Out of these eligible participants, 54 were eventually recruited for the present study, having at least 200 µL of CSF and 200 µL of serum still available for analysis at the time of study design. For each participant, data on medical history and sociodemographic characteristics were collected, including place of birth, residence, and educational attainment. These participants were followed up every 6 months until June 30, 2021, a longer period compared with the prior study, using the criteria for dementia diagnosis previously reported in detail^43^. Briefly, we used Peterson’s criteria revisions proposed by Winblad et al. either for the definition of MCI amnestic form (single domain or multiple domain) or the MCI non-amnestic form of non-vascular origin^54,55^. At each visit, each participant was classified as ‘stable’ or as ‘converter’ to any dementia subtype, including Alzheimer’s dementia^56^, dementia with Lewy bodies^57^, and frontotemporal dementia^58,59^. The diagnoses were systematically a posteriori re-assessed and harmonized according to the most recent and currently accepted diagnostic criteria, such as for Alzheimer’s dementia the CSF biomarkers indicating amyloid deposition, neurofibrillary tangles (phosphorylated Tau) and neurodegeneration (total Tau protein).

***Ethical approval.***

Each participant provided written informed consent to use CSF and blood samples for scientific research purposes, in agreement with the protocol approved by the Modena Ethics Committee (No. 84/2015). The study was conducted in compliance to the guidelines described in the Helsinki Declaration for research involving human participants.

### Analytical determinations

For the collection of CSF and serum samples, lumbar punctures and venepunctures were performed in the morning in fasting participants. Standard international procedures for sample biobanking were followed^60^. Samples were collected in sterile polypropylene tubes and transported to the adjacent laboratory within 30 min from collection. CSF and serum samples were then centrifuged at room temperature at 2700 g for 15 min. and 2000 g for 20 min., respectively, immediately aliquoted in polypropylene vials, anonymized with an alphanumeric code, and stored at − 80 °C until further analysis. Each sample was later transported deep frozen on dry ice by air courier to the Laboratory of Molecular Biology and Metabolism of Tohoku University and kept frozen until the determination of selenoprotein P concentrations was performed.

For measurement of CSF and serum selenoprotein P concentrations, sandwich ELISA system with different capture antibodies was used, keeping laboratory personnel blinded to patient’s information^61,62^. In the BD1 method to determine both full-length selenoprotein P and N-terminal fragment, clone BD1 antibody, recognizing selenoprotein P N-terminal region, was used for capture antibody, and clone AH5, recognizing N-terminal region of selenoprotein P, was labeled with horseradish peroxidase (HRP) and used for the detection antibody. In the AA3 method to measure full-length selenoprotein P, clone AA3, recognizing the C-terminal region, and AH5 conjugated with HRP were used for the capture and detection antibody, respectively. These antibodies were prepared as previously described^61^. Human selenoprotein P was purified from human plasma by using polyethylene glycol, heparin-Sepharose CL-6B column, Q-Sepharose Fast Flow, and Ni–NTA-agarose, respectively^44^. Human frozen plasma was provided by the Japanese Red Cross Tohoku Block Blood Center (No. 25J0012). Ninety-six-well microtiter plates were coated for 18 h at 4 °C with 100 μL of rat anti-human selenoprotein P monoclonal antibody BD1 or AA3 (5 μg/mL) in 0.05 M sodium bicarbonate buffer, pH 9.6, filtrated before use. The dispensed stock solution of each antibody (20–30 mg/mL) was stored at − 30 °C. The wells were washed four times with PBS containing 0.05% Tween 20 (200 µL), and incubated at 37 °C with 150 μL of PBS containing Block Ace (UK-B80, KAC, Japan) for 1 h. After washing the wells four times, 50 μL of selenoprotein P standard or CSF or serum sample (diluted with PBS, containing 0.05% Tween 20 and 0.1% bovine serum albumin, PBS-Tween-BSA) was added to each well, and incubated at 37 °C for 1 h. After washing the wells four times, 50 μL of HRP-conjugated rat anti-human selenoprotein P monoclonal antibody AH5 (20 μg/mL) was added, and incubated at 37 °C for 1 h. Finally, the plates were washed eight times, and dried by shaking. Fifty μL of TMB Peroxidase Substrate (5120-0047, SeraCare Life Science) were added to each well, and the protein-substrate reaction was allowed to proceed for 10 min in the dark. The reactions were stopped by the addition of 50 μL of 1 M sulphuric acid to each well. The absorbances were read at 450 nm in a SpectraMax iD5 (Molecular Devices). We determined at least four points in each CSF or serum sample, and the average value was used for analysis. This assay was shown to be highly reproducible when identical samples are compared in either intra-assay or inter-assay, with the relative standard deviations within determinations being under 5% and 10%, respectively.

Finally, we used analytical determinations of selenoprotein P-bound selenium in the CSF samples, as determined in a previous study in this cohort, using ion exchange chromatography coupled with plasma dynamic reaction cell mass spectrometry^43^.

### Data analysis

We assessed differences in selenoprotein P concentrations in CSF and serum according to participants’ characteristics. We assessed median and interquartile range (IQR) levels for AA3 and BD1 in both CSF and serum according to sex, age category (< 65 and ≥ 65 years), and education (< 8, 8–12, and ≥ 13 years). We also performed a linear and a cubic spline regression analysis of the association between the two selenoprotein P biomarkers, BD1 and AA3, in both blood and CSF, between concentrations of each biomarker in the two different compartments, and between CSF concentrations of selenoprotein P and of selenoprotein P-bound selenium as previously determined in study participants^43^.

We defined person-time at risk as the time between MCI diagnosis and June 20, 2021, or the date of dementia diagnosis, whichever occurred first; the event was defined as the occurrence of dementia (including any dementia subtype, i.e., Alzheimer’s dementia, frontotemporal dementia, dementia with Lewy bodies, vascular dementia). We then estimated the hazard ratio (HR) and 95% confidence interval (CI) of progressing to dementia using a Cox proportional hazards regression model. The model was fitted after assessing all variables for the proportional hazards assumption and adjusting for sex, age at entry as continuous variable, and education. We also computed the hazard ratio in subgroup analysis by restricting the outcome to Alzheimer’s dementia or non-Alzheimer’s dementia, after censoring participants at date of disease diagnosis. The Cox model was complemented by a restricted cubic spline with 3 knots at fixed percentiles (10th, 50th, and 90th), in order to assess non-linear associations between selenoprotein P concentrations and disease risk. Median levels of AA3 and BD1 in CSF (28.69 and 76.00 ng/mL, respectively) and serum (6952 and 10,999 ng/mL, respectively) were used as referent in the Cox model. Secondary analyses were then conducted after excluding cases that progressed to dementia within the first two years of follow-up.

## Supplementary Information

Supplementary Information.

## Data Availability

The dataset for the current study is not publicly available due to privacy restrictions imposed by the ethics committee, as the informed consent obtained from the participants did not include provision for publicly sharing data. However, a minimal and de-identified dataset may be available from the corresponding author upon reasonable request.
