# Supplementary material for: Selenoprotein P concentrations and risk of progression from mild cognitive impairment to dementia
Source: Sci Rep. 2023 May 31;13:8792. doi: 10.1038/s41598-023-36084-6 (PMC10232449; doi:10.1038/s41598-023-36084-6)
Supplement: Supplementary file 1 — Supplementary Information. [file 41598_2023_36084_MOESM1_ESM.docx]

**SUPPLEMENTARY MATERIAL**

**Table S1.** Median (50^th^) and interquartile range (IQR) cerebrospinal fluid levels of amyloid beta, total and phosphorylated Tau in the 54 study participants.

| Biomarker^a^ | Amyloid beta (pg/mL) | Phosphorylated Tau (pg/mL) | Total Tau (pg/mL) |
| --- | --- | --- | --- |
| *All (n=54)* | 600.5 (441.0-806.0) | 68.5 (49.0-88.0) | 337.5 (219.0-614.0) |
| *MCI not converting (n=19)* | 789.0 (521.0-1021.0) | 53.3 (46.0-82.0) | 255.0 (164.0-370.0) |
| *MCI converting to Alzheimer’s dementia (n=26)* | 506.0 (406.0-534.0) | 86.9 (62.0-128.0) | 645.5 (305.0-769.0) |
| *MCI converting to dementia other than Alzheimer’s dementia (n=9)* | 760.0 (686.0-870.0) | 60.0 (48.0-68.0) | 260.0 (221.0-374.0) |

^a^Biomarker cut-off values used at the Modena laboratory: Amyloid beta 1-42 557 pg/mL; Total Tau 350 pg/mL; Phosphorylated Tau181 62 pg/mL).

**Figure S1.** Linear regression analysis for the association between cerebrospinal fluid (CSF) and serum concentrations of AA3 and BD1, serum and CSF concentrations of AA3 and of BD1, CSF selenoprotein P-bound selenium and CSF AA3, and CSF selenoprotein P-bound selenium and CSF BD1. Crude model is represented by dotted line; multivariable model (adjusted for sex, age, and education) is represented by continuous line in 54 individuals with mild cognitive impairment. β represents linear regression coefficient (with 95% C.I.) for the multivariable-adjusted model.

**Figure S2.** Boxplots for BD1 and AA3 cerebrospinal fluid (A) and serum (B) levels according to diagnosis at follow-up.

A)

B)

**Figure S3.** Linear regression analysis for the association between cerebrospinal fluid (CSF) and serum concentrations of BD1 with β-amyloid1-42, total and phosphorylated Tau CSF levels. Crude model is represented by dotted line; multivariable model (adjusted for sex, age, and education) is represented by continuous line in 54 individuals with mild cognitive impairment. β represents linear regression coefficient (with 95% C.I.) for the multivariable-adjusted model.

**Figure S4.** Linear regression analysis for the association between cerebrospinal fluid (CSF) and serum concentrations of AA3 with β-amyloid1-42, total and phosphorylated Tau CSF levels. Crude model is represented by dotted line; multivariable model (adjusted for sex, age, and education) is represented by continuous line in 54 individuals with mild cognitive impairment. β represents linear regression coefficient (with 95% C.I.) for the multivariable-adjusted model.

**Figure S5.** Spline regression analysis for the association between cerebrospinal fluid (CSF) and serum concentrations of BD1 with β-amyloid1-42, total and phosphorylated Tau CSF levels. The solid line represents the multivariable adjusted analysis (adjusted by sex, age, and education) with upper and lower confidence interval showed by shaded area.

**Figure S6.** Spline regression analysis for the association between cerebrospinal fluid (CSF) and serum concentrations of AA3 with β-amyloid1-42, total and phosphorylated Tau CSF levels. The solid line represents the multivariable adjusted analysis (adjusted by sex, age, and education) with upper and lower confidence interval showed by shaded area.

**Figure S7.** Restricted cubic spline analysis of Cox proportional hazards model for the association between baseline selenoprotein P concentrations and risk of developing Alzheimer’s dementia, based on the 26 participants who progressed to dementia after the first 24 months of follow-up (A-D). The solid line represents the multivariable hazard ratio (adjusted by sex, age, education, and APOE4 status) with upper and lower confidence interval showed by shaded area.
